# Understanding Public Opinion in Debates over Biomedical Research: Looking beyond Political Partisanship to Focus on Beliefs about Science and Society

**DOI:** 10.1371/journal.pone.0088473

**Published:** 2014-02-18

**Authors:** Matthew Nisbet, Ezra M. Markowitz

**Affiliations:** 1 American University, School of Communication, Washington, DC, United States of America; 2 Earth Institute and Center for Research on Environmental Decisions, Columbia University, New York, New York, United States of America; 3 Princeton Institute for International and Regional Studies, Princeton University, Princeton, New Jersey, United States of America; University of Pennsylvania, United States of America

## Abstract

As social scientists have investigated the political and social factors influencing public opinion in science-related policy debates, there has been growing interest in the implications of this research for public communication and outreach. Given the level of political polarization in the United States, much of the focus has been on partisan differences in public opinion, the strategies employed by political leaders and advocates that promote those differences, and the counter-strategies for overcoming them. Yet this focus on partisan differences tends to overlook the processes by which core beliefs about science and society impact public opinion and how these schema are often activated by specific frames of reference embedded in media coverage and popular discourse. In this study, analyzing cross-sectional, nationally representative survey data collected between 2002 and 2010, we investigate the relative influence of political partisanship and science-related schema on Americans' support for embryonic stem cell research. In comparison to the influence of partisan identity, our findings suggest that generalized beliefs about science and society were more chronically accessible, less volatile in relation to media attention and focusing events, and an overall stronger influence on public opinion. Classifying respondents into four unique audience groups based on their beliefs about science and society, we additionally find that individuals within each of these groups split relatively evenly by partisanship but differ on other important dimensions. The implications for public engagement and future research on controversies related to biomedical science are discussed.

## Introduction

Over the past decade, there has been considerable research and growing popular interest in what the U.S. National Academies calls the “science of science communication.” [1] Social scientists across fields have investigated the social and cognitive factors that shape public perceptions and opinions about science and technology. Among the major conclusions of this research is that in science-related policy debates, science literacy is only one factor among several that influence public attitudes [2]. The influence of knowledge also often varies by way of an individual's social identity, such that highly knowledgeable and well-educated individuals from different social groups tend to be the most polarized in their opinions. These differences by social group have been observed in studies of public opinion related to stem cell research [3],[4], nanotechnology [5],[6], genetic testing [7], climate change [8], and other topics.

Research in this area not only helps us understand how public opinion is influenced by various political strategies and social factors, but can also inform the communication and outreach efforts of scientists and their institutions. In this regard, social scientists have argued that effective communication depends on conveying the personal and social relevance of a problem or issue while fitting information to the existing values, mental models, experience, and interests of an intended audience [9]. Yet, in the United States, popular discussion of science communication research continues to focus relatively narrowly on differences in public opinion related to political partisanship and on blaming political leaders and the news media for these differences.

This emphasis is somewhat understandable given the extreme polarization among elected officials and activists, differences that are consistently communicated to the public by way of journalists, pollsters, and advocates [10],[11]. Researchers, however, have identified cognitive and social processes beyond partisanship that strongly shape public judgments and decisions. For example, social scientists have examined how core beliefs about science and society shape public opinion [12],[13], and how these underlying schema are often activated by way of the frames embedded in media coverage and popular discourse [14]–[16].

In the present study, we analyze the relative influence of political partisanship and science-related schema on the U.S. public's support for embryonic stem cell research. The political visibility and level of campaigning surrounding Federal funding for embryonic stem cell research allows for a unique comparison of the influence between political partisanship and deeper beliefs about science and society on public opinion formation. Relative to the influence of partisan identity and other factors such as religion, ideology and self-rated knowledge, our findings suggest that generalized beliefs about science and society have a substantially stronger impact on individual judgments, with this influence more chronically accessible and less volatile in relation to media attention and focusing events. Implications for the science of science communication and future research on controversies related to biomedical research and other scientific advances are discussed.

### Partisan Cues, Political Identity and Opinion Formation

Past research suggests that when faced with complexity, uncertainty, and limited time and attention, individuals seldom engage in active deliberation, weighing and assessing many sides and sources of information about a policy debate [3]–[7],[9],[17]. Research instead characterizes individuals as “cognitive misers,” who as a general tendency collect only as much information about a complex topic as they think is necessary to reach a decision [18].

In this regard, researchers have studied how partisan cues in the form of slogans, talking points, and political labels make it easier for individuals to reach decisions efficiently, resulting in a form of “limited information rationality” [19]. In addition, individuals with higher levels of education tend to be the most efficient cognitive misers, as they are better at recognizing partisan cues and determining what others like them think, more likely to react to these cues in ideologically consistent ways, and more skilled at offering arguments to support and reinforce their positions [20]. As a consequence, in policy debates where partisan leaders actively communicate their diverging policy views, differences in opinion among college-educated Republicans and Democrats tend to be greater on average than those among their lesser-educated counterparts [10].

Consider the findings of previous studies that have tracked the increasing availability of diverging partisan cues on embryonic stem cell research and the influence on public opinion. In 2001, as President Bush debated a possible ban on government funding for research, Republican Party leaders split on the issue. Some supported funding while others sided with religious groups in opposing funding. Given conflicting cues among Republican leaders, analyses of nationally representative survey data show that in 2001, 2002 and 2003, partisan identification had no statistically significant impact on public support for government funding. Instead, after controlling for a number of confounds, religious identification and beliefs were the strongest influences on public judgments [3],[21].

Yet, in the months leading up to the 2004 presidential election, partisan differences were made readily apparent for the public by way of campaign messaging and news coverage. Democratic campaign strategists viewed stem cell research as a politically favorable “wedge” issue and employed targeted messaging designed to win votes from moderate and weak-identifying Republicans. [19],[21]. As Table 1 indicates, during the election, Americans became increasingly aware of the diverging positions of the two presidential candidates. Among registered voters interviewed before and after the first presidential debate, knowledge of Kerry's support for expanded funding increased by 25% so that two-thirds could correctly place each candidate's position relative to the issue.

**Table 1:** Percentage of U.S. registered voters aware of 2004 presidential candidates' position on funding for research.

	*9/04*	*10/04*	*9/04*	*10/04*
	(%) Bush	(%) Bush	(%) Kerry	(%) Kerry
In Favor	15	12	43	68
Opposed	58	68	9	5
Don't Know	26	19	47	27
*Refused*	*1*	*1*	*2*	*–*
*N*	*425*	*425*	*425*	*425*

Note: Respondents were asked: “As far as you know, is [George W. Bush/John Kerry] in favor or opposed to the use of federal funds for stem cell research which involves the destruction of living embryos?” National sample of registered voters only. The respondents were originally interviewed September 13–29, 2004 and re-interviewed October 14–17 following the presidential debate held on October 13, 2004. Source: HealthPulse survey conducted by Stony Brook University Center for Survey Research.

The ability of the public to readily recognize the differences between the two presidential candidates on stem cell funding stands in contrast to other survey results that measured more complex dimensions of knowledge. In these surveys, even among those saying they were interested in the debate over stem cell research, only a small proportion of respondents correctly knew that adult stem cells had been used in research for years or that fewer than 100 embryonic stem cell lines were eligible to be used in Federally funded studies [22].

Following the 2004 election, analyses of representative survey data showed that religious identity remained a major influence on support for government funding of embryonic stem cell research. However, in contrast to the 2001–2003 period, partisanship also emerged as a substantial predictor of public opinion and policy support. “As elites pulled apart ideologically on this issue,” concluded political scientist Matthew Levendusky, “ordinary voters took their cues from elites and aligned their own party and views on the issue.” [21]

As various other researchers have tracked, stem cell research remained not only a highly salient partisan debate during the 2006 and 2008 elections but was also the subject of campaigning across a number of politically strategic states. These dynamics likely served to strengthen the influence of partisanship on the public's stem cell-related attitudes and policy preferences [3],[4], [14], [23]–[25]. Only following President Obama's 2009 decision to expand government funding did the intensity of campaign efforts and news attention decline [26].

### Framing, Schema and Opinion Formation

In the stem cell debate, as elected officials and activists focused on communicating the differences between candidates and political parties, they also conveyed specific “frames” of reference for why embryonic stem cell research mattered and what was at stake for society. Both advocates for and opponents against Federal funding predominately framed the debate as a moral matter. To convey their reservations about research, those opposed to funding argued that it was morally wrong to destroy embryos, since they constitute human life. They conveyed this meaning by relying on metaphors and catchphrases such as “scientists playing God,” allusions to *Frankenstein*, *Brave New World*, or *1984* [27] and by making moral appeals to the sanctity and purity of human life [25].

In contrast, those advocating for expanded funding emphasized the moral duty to move forward with scientifically promising research that could benefit many Americans. They did so by referencing metaphors such as scientists “racing to find a cure,” arguing that it was “pro-life to be pro-research,” and by emphasizing the many types of diseases and health problems that could be treated with stem cell-derived therapies, thereby highlighting the moral duty to help suffering patients [25], [27]. News coverage of the stem cell debate tended to strongly reflect the framing strategy of those advocating for expanded funding. Coverage at major newspapers and on broadcast TV news emphasized scientific progress and breakthroughs, quoted or mentioned a substantially greater number of research advocates than research opponents [27], and emphasized the moral duty to protect patients from harm [25].

These two contending frames in the stem cell debate—one focusing on moral limits to research and the other on the moral duty to move ahead with research—set the context for public judgments and opinions by selectively activating different cognitive schema. Once activated by a particular frame of reference, schema provide short cuts for reaching an opinion about a complex topic such as stem cell research, serving as a basis for inference, and operating as a mechanism for storing and retrieving information from memory [28]–[30].

Researchers studying public attitudes about science and technology have identified two major schema that individuals rely on to form judgments and generate opinions. The first schema, “scientific optimism,” is an attitude construct representing respect for the intentions of scientists, a sense that science and technology provide useful results and products for society, and the assumption that future benefits from science and technology are likely. The second schema, “scientific reservations,” is an attitude construct reflecting public concerns about the speed of change in modern life and a sense that science and technology pose conflicts with traditional values or belief systems [31],[32],[13].

In the U.S. context, the two schema tend to be negatively correlated with one another. Thus “science optimists,” who hold a strong belief in the promise of science and technology, are generally less likely to have concerns about negative impacts. In contrast, “science pessimists,” who have strong reservations, are less likely to acknowledge the benefits of science and technology to society [33],[13]. In previous studies examining public opinion about biomedical research, even after controlling for partisanship and ideology, those scoring high on scientific reservations were on average more likely to oppose genetic engineering and embryonic stem cell research specifically. In contrast, those scoring high on scientific optimism were more likely to support such advances [33],[14].

Studies also show that some individuals hold both schema strongly and concurrently, perhaps reflecting among this “conflicted” group a more nuanced and complex consideration of the role of science and technology in society [33],[14]. Additionally, some people may score low in both schema which suggests that this “disengaged” group may lack a strong mental model for how science and technology might generally impact their lives and society more broadly. In the absence of clear guiding schema about science, such individuals may be more apt to rely on other heuristics such as partisanship when asked to make judgments about unfamiliar scientific issues or technologies.

History, national identity, and political culture also play important roles in shaping public attitudes about biomedical research and underscore why a focus on schema and framing has broader generalizable value than a more limited focus on partisan differences in U.S. public opinion. For example, survey studies comparing attitudes to embryo research across European countries find that individuals living in Germany, Poland, and Austria are the least supportive of such research. In these countries, with the cultural memory of Nazi-era science and medicine shaping public views, reservations about embryo research span Catholic and Protestant identity as well as levels of science literacy [32].

### Present Research

As reviewed, studies in the field of political communication have focused on the process by which diverging messages from Democratic and Republican political leaders promote polarized differences in public views of complex science-related policy debates. Moreover, given the tendency for college-educated Democrats and Republicans to more efficiently recognize and process these partisan cues, studies show that when political leaders disagree on policy, this segment of the public tends to be more polarized in their attitudes than their non-college educated counterparts.

Research in the field of science communication, however, has focused on more generalized beliefs—or “schema”—about science and society as major factors shaping public views of policy debates. This research across country-setting and cultural context has also emphasized the process by which selective frames of reference about an issue differentially activate one set of schema over another, thereby influencing perceptions.

Yet, despite these two identified pathways to opinion formation in science-related policy debates, to date, only a few studies of U.S. public opinion have compared the relative influence of partisan identity and schematic beliefs about science and society on public opinion and policy preferences. Moreover, given the likely importance of beliefs about science and society, more research is needed on how major demographic groups might differ relative to these schema.

Therefore, in the current study, using nationally representative survey data collected between 2002 and 2010, we first segment Americans by their beliefs about science and society, describing the composition of these groups by key demographic variables. Next, we investigate the relative impact of political partisanship and beliefs about science and society on U.S. public opinion about embryonic stem cell research. Consistent with previous studies, we expect partisanship will have a significant influence on public opinion and that the influence of partisanship will vary by levels of education. Yet even after controlling for partisanship, ideology, religion, self-rated knowledge and other potential predictors, we expect that schema relative to the social implications of science and technology will have a unique and substantial influence on public opinion. We additionally investigate how the influence of schema may vary by level of education.

If schema specific to the social implications of science and technology do have unique and substantial influences on public opinion, then this suggests important implications for looking beyond partisan differences when analyzing future controversies over biomedical research and when planning public communication and engagement strategies, a topic we return to in the conclusion.

## Methods

### Data

For our analysis, we used combined data from the 2002, 2003, 2004, 2005, 2006, 2007, 2008 and 2010 Virginia Commonwealth University (VCU) Life Sciences Surveys, data sets which can be downloaded via the University of Connecticut's Roper Center for Public Opinion Research. (No survey was conducted in 2009.) Across all eight surveys, the pooled data set featured 8,015 unique respondents. Table 2 includes the specific dates that the surveys were conducted and the reported response rates. Interviewers used computer-assisted telephone interviewing software. For all years, the sample of telephone numbers was designed so that all residential phone lines in the U.S. had a known chance of inclusion.

**Table 2:** Dates, sample size, and response rates for VCU Life Sciences Surveys, 2002 to 2010.

Year	N	Month	Response Rate	Cooperation Rate	Survey Firm
2002	1000	9/4–9/16	24%	27%	SERL
2003	1003	9/3–9/26	26%	31%	SERL
2004	1004	9/7–9/17	23%	28%	SERL
2005	1002	9/14–9/29	26%	Not reported	SERL
2006	1000	11/7–11/21	25%	37%	Abt SRBI
2007	1000	11/26–12/9	21%	33%	Abt SRBI
2008	1005	11/24–12/7	25%	32%	PDS
2010	1001	5/12–5/18	15% (land), 20% (cell)	18% (land), 27% (cell)	PDS

Note: Survey data collected by the Virginia Commonwealth University Life Sciences Surveys, 2002–2010. No survey was conducted in 2009. The survey questions were developed jointly by VCU Life Sciences and by the VCU Center for Public Policy. All surveys by the designated survey firms were conducted by either landline or cell phone. The sample of landline and cell telephone numbers was designed so that all residential telephones, including new and unlisted numbers, had a known chance of inclusion. Response rates based on AAPOR RR3 calculation method. The data used in our analysis were weighted to adjust for unequal probabilities of selection due to multiple adults living in the household. In addition, the data are weighted on sex, age, education, race/ethnicity, and region to reflect the demographic composition of the adult population in the U.S. The surveys in 2008 and 2010 were additionally weighted by population density. All percentages reported in the current study's figures and tables are weighted.

The data used in our analysis are weighted to the U.S. population by year. Within year, data were weighted to adjust for unequal probabilities of selection due to multiple telephone lines and multiple adults living in the household; the data were also weighted on sex, race, age, education and region of residence. Due to differences in weighting procedures across years, weights were adjusted so that final, weighted sample sizes were approximately equal across all years (so as to avoid any one year having undue effects on estimates obtained from analyses run with the entire sample).

### Measures

#### Support for embryonic stem cell research

Our dependent variable measuring views of embryonic stem cell research consisted of a single item asking respondents: “On the whole, how much do you favor or oppose medical research that uses stem cells from human embryos - do you strongly favor, somewhat favor, somewhat oppose, or strongly oppose this?” The support for research measure was reverse coded so that higher scores reflect increasing support for research (M_total sample_ = 2.58, *SD* = 1.09; 4-point scale, ‘Strongly oppose’ coded 1 to ‘Strongly favor’ coded 4).

#### Demographics

Demographic variables controlled for in our regression model include educational attainment (71.8% with ‘some college’ or less, 28.2% with four years of ‘college’ or more; some college or less was coded ‘0,’ college or higher was coded ‘1’), sex (52.2% female; male coded ‘0,’ female coded ‘1’), age (M = 45.68 years, *SD* = 17.52), income (M = 3.32, *SD* = 1.44; recoded on 5-point scale from ‘under $20,000’ to ‘greater than $70,000), and race (77.6% White, 22.4% Other; White coded as ‘0,’ Other coded as ‘1’). Our decision to use education as a dichotomous variable reflects previous studies on the important differences in the processing of partisan cues and messages between college and non-college educated individuals.

#### Partisan identity and ideology

Partisan identification was measured using the item, “Do you normally consider yourself a Democrat, a Republican or an Independent?” Across all years in the combined data set, 36.4% of participants identified as Democrats, 29.7% as Republicans and 33.9% as Independents. For our regression analysis, we included dummy codes for Republicans and Independents. Political ideology was measured using the item, “How would you describe your views on most political matters? Do you consider yourself liberal, moderate, or conservative?” Across all years, 22.2% of participants identified as liberal, 41.6% as moderate, and 36.3% as conservative. Ideology was included as a continuous measure in our regression analysis with conservatives coded high.

#### Christian denominational affiliation and religious belief

We also included dummy codes for Christian denominational affiliation, including Protestant (53.7% of respondents), and Catholic (22.6% of respondents). In addition, we included two proxy measures of religious commitment. First, we controlled for frequency of church attendance (M = 3.72, *SD* = 1.61; 6-point scale from ‘Never’ coded ‘1’ to ‘More than once a week’ coded ‘6’). Previous research has found that frequency of church attendance reflects not only personal commitment to a religious institution but is also an indirect measure of exposure to church based communication about a political debate such as stem cell research [34], [35].

Second, we created an index of religious belief by combining a measure that asked how much guidance religion played in a respondent's life with a measure of biblical literalism. This latter question asked respondents whether in their view: “The Bible is the actual Word of God, The Bible is the Word of God but not everything in it should be taken literally, or the Bible is a book written by men and is not the Word of God?” Responses to these two items were standardized and summed (α = .70), such that higher scores indicate greater religiosity (M = .05, *SD* = 1.71, range −3.39 to 1.98). In past research, these items in combination have been shown to capture the type of strong doctrinal conservatism [36] that shapes opinion and preferences on stem cell research [13], [14].

#### Abortion beliefs

Also, consistent with previous research analyzing public opinion on stem cell research [14], abortion-related beliefs were controlled for using a standard item that asked: “Which of these comes closest to your views about abortion? A woman should be able to get an abortion if she decides she wants one no matter what the reason, abortion should only be legal in certain circumstances, such as when a woman's health is endangered or when the pregnancy results from rape or incest, or abortion should be illegal in all circumstances.” The most restrictive views on abortion allowed by the question (illegal in all circumstances) was coded ‘1,’ and the moderate and most permissive positions (‘legal in certain circumstances,’ ‘no matter what reason’) were coded ‘0’ (16.3% ‘always illegal’).

#### Self-rated knowledge about scientific and medical research

Direct measures of science knowledge and literacy were not available as part of the VCU surveys. However, items tapping respondents' own assessment of how informed they were about science and medicine were available. Self-assessed knowledge consisted of two items (r = .72) in which respondents were asked how informed they were about new scientific discoveries and how informed they were about new medical discoveries. The two items were averaged (M = 2.78, *SD* = .61), with higher scores on this measure indicating increasing levels of self-rated knowledge.

#### Schema related to science and society

To measure “scientific reservations” about the impact of science and technology on society, participants were asked how much they agreed with the following statements: ‘Scientific research these days doesn't pay enough attention to the moral values of society’ (M = .59, *SD* = .33) and ‘Scientific research has created as many problems for society as it has solutions’ (M = .52, *SD* = .32). To improve comparability between the schema measures and the party identification dummy variables in subsequent multiple regression models, the two items were coded on a 0 to 1 scale (strongly disagree was coded as ‘0,’ disagree as ‘.33,’ agree as ‘.67’ and strongly agree as ‘1’). The two items were combined (r = .38) to form a single measure of individuals’ schema related to reservations about the impact of scientific research on society.

To measure schema related to “scientific optimism,” participants were also asked how much they agreed with the following statements (same four-point scale, same coding as above): ‘Scientific research is essential for improving the quality of human lives’ (M = .83, *SD* = .24) and ‘New technology used in medicine allows people to live longer and better’ (M = .83, *SD* = .24). These items were combined (r = .40) to create a single measure of individuals' schema related to the social promise of scientific research.

The composite measures of scientific optimism and scientific reservations were negatively correlated, *r* = −.26, *p*<.001, as expected.

#### Interaction terms

Interaction terms were constructed in order to explore the possible moderation effect of education level on both partisan identity and schema. These measures enable us to examine how the effects of partisanship and schema might vary between college-educated and non-college educated respondents. To do so, dummy codes for Republicans and Independents were multiplied by the dichotomous measure of educational attainment to create the first set of interaction terms. To create interaction terms between education and scientific reservations and scientific optimism, respectively, the reservations and optimism measures were first centered and then separately multiplied by the education variable.

### Analytical Procedure

To better inform our multivariate analysis of relative influences on public opinion, we began by examining how the U.S. public differs in their more generalized, schematic views about science and society, constructing a typology of respondent types. To do so, using the pooled data set, we first submitted the two items comprising the “scientific reservations” schema and the two items comprising the “scientific optimism” schema to a principle components analysis using oblique rotation. Based on the past studies reviewed, our assumption was that the two constructs would be negatively correlated with each other.

We extracted two negatively correlated principle components (r = −.28). Together, the two rotated factors explained 69.63% of the variance in the four items. All four items loaded above .80 on their expected component and all cross-loadings were negative and less than .30. Four respondent types were then defined and participants assigned appropriately by crossing scores on the resulting two factors. Participants who scored high on scientific optimism and low on scientific reservations were categorized as “Scientific Optimists” (35.9% of sample). Those who scored high on scientific reservations and low on scientific optimism were categorized as “Scientific Pessimists” (23.3% of sample). Respondents who scored high on both measures were categorized as “Conflicted” (24.6% of sample). Those respondents who scored low on both the “reservations” and “promise” factors were categorized as “Disengaged,” signifying that they lacked a strong mental model relative to the social implications of science and technology (16.2% of sample).

Next, ordered probit regression was employed to examine the relative impact of various independent variables on participants' support for stem cell research. Due to the limited number of level-2 units included in the complete dataset (i.e., eight years of data), we did not formally model change or stability over time in public opinion related to embryonic stem cell research (e.g., by using multilevel models to examine cross-level interactions). Instead we carefully restricted our formal analysis to the level at which our data were collected, namely, the individual. Differences across years were controlled for by first entering dummy variables for each year (2002 served as the reference). We ran a series of nested models, in which our primary variables of interest (i.e., party identification and science schema) were entered in separate blocks after all other independent variables had been included in the model. This allowed us to look specifically at the improvements in model fit due to these two sets of variables.

Following established methodological procedures, we additionally used data visualization techniques to qualitatively explore trends over time relative to differences in attitudes by partisanship and schema [37].

## Results

**Figure 1:**
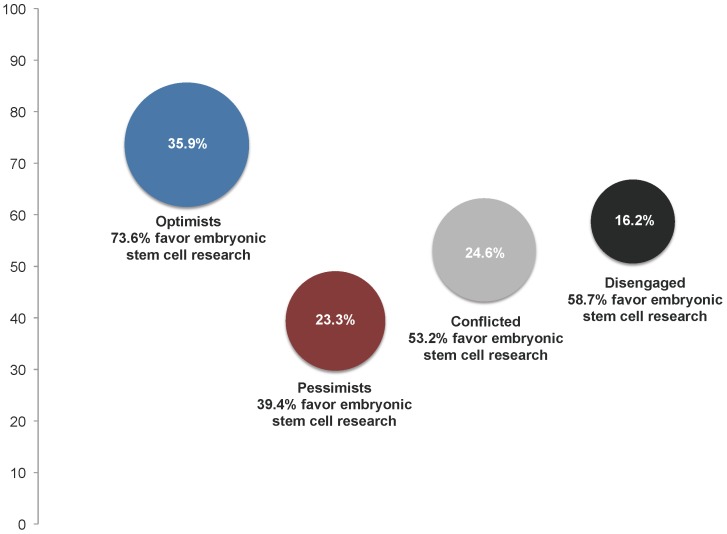
Views on science and society as a proportion of U.S adult population and by percentage favoring embryonic stem cell research. Unique audience segments relative to their views on the social implications of science and society were identified using principle components analysis, oblique rotation. Participants who scored high on scientific optimism and low on scientific reservations were categorized as “Scientific Optimists” (35.9% of respondents). Those who scored high on scientific reservations and low on scientific optimism were categorized as “Scientific Pessimists” (23.3% of sample). Respondents who scored high on both measures were categorized as “Conflicted” (24.6% of sample). Those who scored low on both measures were categorized as “Disengaged” (16.2% of sample). Size of the bubbles for each schema-related audience segment are proportional to the percentage of relevant respondents within the pooled, aggregated data sets (N = 8,105). To measure their views on embryonic stem cell research, respondents were asked: “On the whole, how much do you favor or oppose medical research that uses stem cells from human embryos - do you strongly favor, somewhat favor, somewhat oppose, or strongly oppose this?” Source: Virginia Commonwealth University Life Sciences Surveys, 2002–2010. No survey was collected in 2009.

Table 3 presents the four segments based on respondents' views of science and society, highlighting their composition in terms of major demographic variables and their preferences regarding embryonic stem cell research. (These four segments were derived using the previously described principal components analysis.) Based on data from the pooled sample across years, Figure 1 plots each segment relative to their support for embryonic stem cell research. Bubbles represent the proportion of each segment within the population.

**Table 3:** Major characteristics of U.S. adults as segmented by their views on science and society.

Category	Subcategory	Optimists	Pessimists	Conflicted	Disengaged	Significance
Percent of total		35.9	23.3	24.6	16.2	
Sex (%)	Female	50.3	54.8	54.0	49.9	?^2^ = 13.3
	Male	49.7	45.2	46.0	51.1	
Education (%)	HS or less	33.2	52.6	51.5	45.4	?^2^ = 284.9
	Some college	27.6	25.9	26.6	25.2	
	College	19.8	12.8	12.2	15.0	
	Post-College	19.4	8.7	9.7	14.4	
Income (%)	<$20,000	10.6	16.9	14.8	16.2	?^2^ = 200.7
	20,000–35,000	13.1	23.7	17.5	17.1	
	35,000–50,000	18.5	18.6	20.5	17.6	
	50,000–70,000	17.7	18.5	20.3	18.2	
	>70,000	40.2	22.4	26.8	31.0	
Race/Ethnicity (%)	White	82.3	73.4	76.7	75.7	?^2^ = 52.1
	Other	17.7	26.4	23.3	24.3	
Age (median)		43	42	47	40	F(3, 7070) = 27.4
Party ID (%)	Republican	30.2	30.2	33.6	23.9	?^2^ = 52.8
	Independent	31.4	37.8	30.9	38.0	
	Democrat	38.4	32.0	35.5	38.0	
Ideology (%)	Liberal	25.7	17.5	19.2	27.0	?^2^ = 156.5
	Moderate	45.8	36.8	40.1	39.4	
	Conservative	28.5	45.7	40.7	33.6	
Abortion (%)	Legal	90.6	74.8	83.3	83.1	?^2^ = 181.0
	Illegal	9.4	25.2	16.7	16.9	
Self-rated knowledge		2.9	2.7	2.8	2.7	F(3, 7192) = 50.5
ESC support (%)	Yes	73.6	39.4	53.2	58.7	?^2^ = 475.1
	No	26.4	60.6	46.8	41.3	

Note: All χ^2^ and F tests are significant at p<.01. Source: Virginia Commonwealth University Life Sciences Surveys, 2002–2010, N = 8,105. No survey was collected in 2009.

Examining the aggregated data from the eight surveys, 74% of **Scientific Optimists** say they either strongly favor or favor embryonic stem cell research. Members of this segment are disproportionately white, have the highest incomes and average educational level with 40% holding at least a four-year college degree. They tend to split almost evenly by partisan identity though trend slightly more Democrat. In terms of ideology, they are the most moderate in their outlook and almost all believe that abortion should be legal. Specific to self-rated knowledge, among the four segments, they tend to consider themselves the best informed about science and medicine.

Among **Scientific Pessimists**, only 39% say they either strongly favor or favor embryonic stem cell research. Individuals in this segment tend to be the least well educated with 78% lacking a four-year college degree and tend to earn the lowest incomes with 40% earning less than $35,000 a year. This group also has the highest proportion of non-whites (26%) and the highest proportion of women (55%). Scientific Pessimists split evenly relative to partisan identity but tend to be disproportionately either moderate (37%) or conservative (46%) in their ideological outlook. Among this group, roughly 1 in 4 believes that abortion should be illegal no matter the circumstance. Interestingly, this group also tends to view themselves as the least informed about science and medicine.

Among the **Conflicted**, 53% say that they either strongly favor or favor embryonic stem cell research. In terms of education, gender, and income this segment is similar in composition to the Scientific Pessimists and is also the oldest of the four segments. The Conflicted on the whole are slightly more Democrat but are also more moderate and conservative in their political outlook. More than 8 out of 10 believe that abortion should be legal. Despite lower levels of education, they tend to rate themselves relatively high in terms of knowledge about science and medicine.

Among the **Disengaged**, 59% say that they either strongly favor or favor embryonic stem cell research. Next to Scientific Optimists, this group has the highest income and roughly 30% have at least a four-year degree. These individuals tend to lean Democrat or Independent and lean moderate in their ideological outlook. They have a similar level of support for abortion as the Conflicted. In comparison to Scientific Optimists, a distinguishing trait is their lower levels of self-rated knowledge about science and medicine. Given that this group lacks a well-developed mental model for the social implications of science and consider themselves less informed, it is likely that the Disengaged are therefore more reliant on their partisanship to guide their judgments. The higher levels of support among this group for stem cell research, along with their tendency to lean more Democrat and Independent, is suggestive of this likelihood.

### Testing the Relative Influence of Partisanship and Beliefs about Science and Society

To test the relative influence of political partisanship and schema about science and society on public support for embryonic stem cell research, we ran a series of ordered probit regression models testing the unique effects of these variables while also controlling for other plausible influences. Results are presented in Table 4. The first column shows results from our base model (“Covariates Only”), which included the dummy variables for time, the demographic predictors (age, sex, education, income, race) as well as religious affiliation, church attendance, strength of religious belief, abortion beliefs, and self-rated knowledge about scientific and medical research. In the second regression (“Party Id”), each of these variables were included plus dummy variables for Republican and Independent partisan identification. In the third regression (“Schema”), each of these previous variables were included and the measures of Scientific Reservations and Scientific Optimism were added.

**Table 4:** Results of ordered probit regression models predicting U.S. adult support for embryonic stem cell research.

Predictor	Covariates only		Party Id		Schema	
	Estimate	*SE*	Estimate	SE	Estimate	SE
Year						
2003	.219***	.*062*	.218***	.*062*	.196**	.*062*
2004	.442***	.*062*	.437***	.*062*	.406***	.*063*
2005	.479***	.*063*	.469***	.*063*	.450***	.*064*
2006	.310***	.*060*	.306***	.*060*	.264***	.*061*
2007	.286***	.*061*	.285***	.*061*	.272***	.*062*
2008	.373***	.*062*	.359***	.*062*	.318***	.*063*
2010	.446***	.*062*	.440***	.*062*	.412***	.*063*
Age	.006***	.*001*	.006***	.*001*	.005***	.*001*
Sex	.052∧	.*031*	.033	.*031*	.036	.*032*
Education	.152***	.*036*	.153***	.*037*	.078*	.*037*
Income	.045***	.*012*	.055***	.*012*	.034**	.*012*
Race	.034	.*039*	−.036	.*040*	.034	.*041*
Ideology	−.315***	.*022*	−.263***	.*023*	−.230***	.*023*
Protestant	−.008	.*045*	−.014	.*045*	−.036	.*045*
Catholic	−.097*	.*048*	−.116*	.*049*	−.145**	.*049*
Church attendance	−.092***	.*013*	−.091***	.*013*	−.080***	.*013*
Religious belief	−.174***	.*012*	−.169***	.*012*	−.154***	.*012*
Abortion beliefs	−.743***	.*046*	−.734***	.*046*	−.670***	.*047*
Self-rated knowledge	.277***	.*027*	.278***	.*027*	.227***	.*028*
Republican			−.284***	.*043*	−.293***	.*043*
Independent			−.178***	.*038*	−.153***	.*038*
Reservations					−.928***	.*062*
Optimism					.854***	.*085*
Threshold 1	.162	.114	−.004	.*118*	−.242	.*120*
Threshold 2	.816	.114	.653	.*118*	.443	.*120*
Threshold 3	1.975	.116	1.818	.*119*	1.661	.*122*
Nagelkerke R^2^	.327		.333		.387	
−2* Log Likelihood	12654.3		12609.9		12139.6	
Δ χ^2^ (DF)	1960.2 (19)***		44.4 (2)***		470.3 (2)***	

Note:

* p<.05.

** p<.01.

*** p<.001.

Respondents were asked: “On the whole, how much do you favor or oppose medical research that uses stem cells from human embryos - do you strongly favor, somewhat favor, somewhat oppose, or strongly oppose this?” Responses are coded in the direction of increasing support. Source: Virginia Commonwealth University Life Sciences Surveys, 2002–2010, N = 8,105. No survey was collected in 2009.

Regression coefficient estimates are reported, along with standard errors and model fit statistics. As reported in Table 4, the “Party Id” model resulted in a significant improvement over the base model, χ^2^ = 44.2, df = 2, p<.001. Moreover, the “Schema” model was significantly better fitting than the nested “Party Id” model, χ^2^ = 470.3, df = 2, p<.001. Looking at both coefficient estimates and model fit statistics, it is clear that the two schema measures were much stronger unique predictors of stem cell support than partisan identification.

Among demographic variables in the first and second regression, education was significantly related to support. However, after schema were included in the third model, the effect of education was attenuated, suggestive of a possible mediation effect. Among other demographic variables, after all variables are entered in the model, older Americans were slightly less likely to support research and higher wage earners more likely to do so. This latter finding is perhaps reflective of stronger ties to the marketplace among higher wage earners, with these individuals viewing embryonic stem cell research as a new target for investment or offering future treatments (that would be affordable for their income bracket).

In terms of the influence of religion on support for embryonic stem cell research, in the third regression, after all controls, Catholics were appreciably less likely to support research (compared to other denominations), as were those who attended church more frequently and those who held stronger overall religious beliefs. Consistent with previous research [14], an individual's pre-existing attitude regarding abortion was among the strongest influences in the model after controlling for other factors. Also consistent with past research, those respondents who considered themselves well informed about science and medicine were also more likely to favor embryonic stem cell research [14].

In terms of political partisanship and ideology, in the third model, taking into account all previous and subsequent predictors, both Republicans and Independents were less likely than Democrats to favor embryonic stem cell research. Similarly, after controls, those who scored higher in terms of political conservatism were also less likely to support research.

Finally, schema related to scientific reservations and scientific optimism were the strongest unique predictors in the model. Those scoring higher on scientific reservations were significantly less likely to support embryonic stem cell research and those scoring higher on scientific optimism were significantly more likely to support such research, as predicted. Of particular interest to the present research, beliefs about science and society held the strongest overall unique relationships to public support for embryonic stem cell research, controlling for and in comparison to partisan identity, ideology, religion, and abortion views.

### Moderating Effects of Education

Next, in order to formally test how the influence of partisan identity may vary as a function of educational attainment (as has been previously been demonstrated in past studies), the interactions between party identification and educational attainment were entered in a fourth model. As shown in Table 5, both the interaction between Republican (versus Democratic) identity and education, as well as the interaction between Independent (versus Democratic) identity and education, were significant (see first model of Table 5, showing only coefficients for the interactions). This indicates that the effect of partisanship on support for embryonic stem cell research is stronger among the higher educated than among those of lower educational attainment. More specifically, the difference in support for stem cell research between college educated Republicans and college educated Democrats (and between college-educated Independents and college educated Democrats) is larger than the difference in support among non-college educated Republicans, Independents and Democrats.

**Table 5:** Results of ordered probit regression models testing interactions between education and partisanship and education and science schema.

Predictor	Party Interaction		Schema Interaction	
	Estimate	*SE*	Estimate	*SE*
Moderating effects				
Republican X Education	−.267**	.*082*		
Independent X Education	−.174*	.*083*		
Reservations X Education			−.573***	.*129*
Optimism X Education			.178	.*205*
Threshold 1	−.195	.122	−.263	.120
Threshold 2	.490	.122	.422	.120
Threshold 3	1.709	.123	1.644	.122
Nagelkerke R^2^	.388		.389	
−2* Log Likelihood	12128.2		12119.3	
Δ χ^2^ (DF)	11.1 (2)**		20.3 (2)***	

Note:

* p<.05.

** p<.01.

*** p<.001.

In testing each of the above interactions, all previous variables displayed in Table 4 are controlled for (not shown for clarity). The interactions testing education and partisanship and the interactions testing education and schema were tested independently of each other by entering them as the final variables in separate regressions. Model improvement statistics are calculated relative to the final model shown in Table 4.

**Figure 2:**
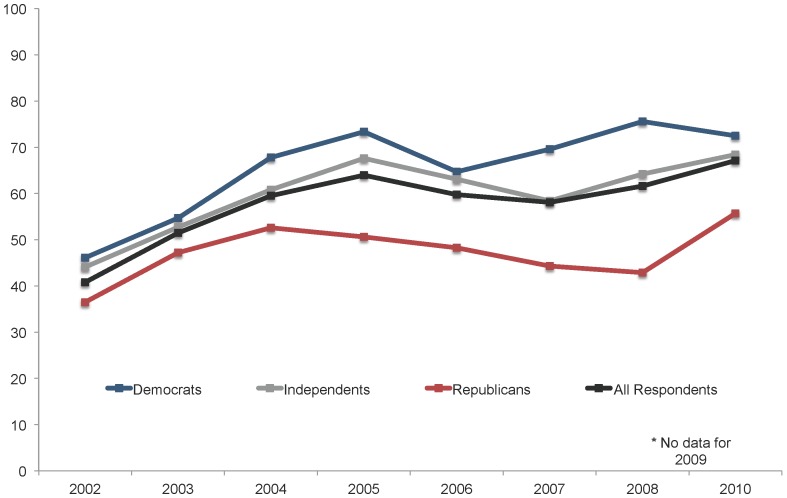
Percentage of U.S. adults by partisanship who favor embryonic stem cell research, 2002–2010. Respondents were asked: “On the whole, how much do you favor or oppose medical research that uses stem cells from human embryos - do you strongly favor, somewhat favor, somewhat oppose, or strongly oppose this?” Source: Virginia Commonwealth University Life Sciences Surveys, 2002–2010. No survey was collected in 2009.

In order to explore qualitatively how these relationships may have changed or remained constant across years, we first plotted the proportion of all VCU survey respondents, regardless of education level, who said they either strongly favored or favored embryonic stem cell research (see Figure 2). Levels of support are shown separately for self-identified Democrats, Republicans and Independents for each of the eight years for which we have data. As suggested by previous studies of the issue, the gap in views on embryonic stem cell research between Democrats and Republicans appeared to increase following the 2004 election.

**Figure 3:**
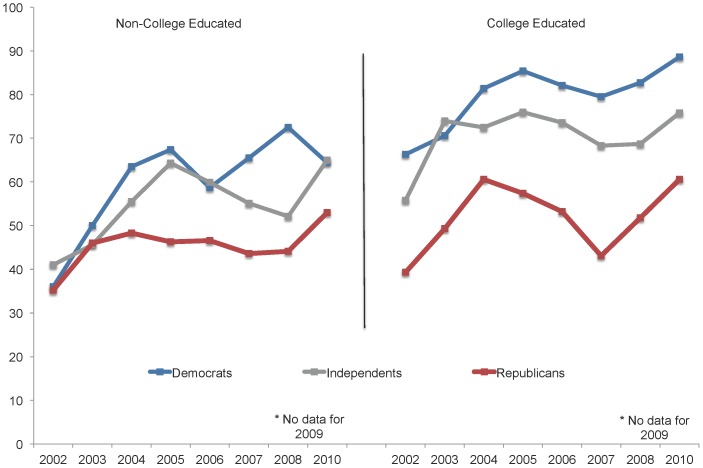
Percentage of U.S. adults by partisanship and education who favor embryonic stem cell research, 2002–2010. Respondents were asked: “On the whole, how much do you favor or oppose medical research that uses stem cells from human embryos - do you strongly favor, somewhat favor, somewhat oppose, or strongly oppose this?” College-educated adults include those with a four-year college degree or higher. Non-college educated adults include those with a two-year associates degree, some college experience, a high school degree, or less. Source: Virginia Commonwealth University Life Sciences Surveys, 2002–2010. No survey was collected in 2009.

Next, we plotted support for embryonic stem cell research among non-college educated and college educated Republicans, Independents, and Democrats. As can be seen in Figure 3, as expected, differences in support for stem cell research between Republicans and Democrats differed in magnitude as a function of education, with a larger gap observed among those individuals with at least a four year college degree. As early as 2002, the more politically attuned college-educated partisans were already strongly split in their views on embryonic stem cell research. However, it was not until after the 2004 election that their non-college educated counterparts began to exhibit similar partisan cleavages and even then they consistently show less separation than their better-educated counterparts.

In a final model, we tested the interactions between education and the two schema measures, reservations and optimism. Similar to the interaction we observed with partisanship, this allowed us to investigate whether better-educated individuals were more adept at applying their beliefs about the social implications of science and technology to the stem cell debate. As our results show in Table 5, there was a relatively strong interaction between scientific reservations and education but no significant interaction between education and scientific optimism. This indicates that among better educated members of the public, reservations about the impact of science on society have a stronger (and more negative) influence on support for embryonic stem cell research than among the less well educated.

**Figure 4:**
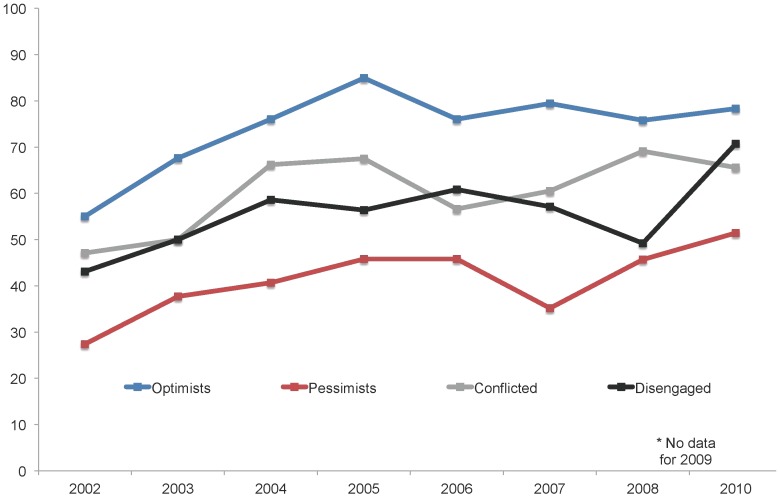
Percentage of U.S. adults by views on science and society who favor embryonic stem cell research, 2002–2010. Respondents were asked: “On the whole, how much do you favor or oppose medical research that uses stem cells from human embryos - do you strongly favor, somewhat favor, somewhat oppose, or strongly oppose this?” Source: Virginia Commonwealth University Life Sciences Surveys, 2002–2010. No survey was collected in 2009.

In Figure 4, we return to the previously identified audience segments relative to views of science and society to qualitatively explore how support for embryonic stem cell research among these groups may have shifted across years. Scientific Optimists across years were, unsurprisingly, the most supportive of research and grew increasingly so through 2005, peaking that year at 84.9% in favor. The proportion favoring research dipped across the next few years, registering at 78.3% in 2010. Interestingly, Scientific Pessimists moved from only 27.4% support in 2002 to 51.4% favoring research in 2010.

The Disengaged shifted from 43.1% in favor in 2002 to 70.7% support by 2010. This group also appeared to be the most volatile in their outlook, declining 11% in their support between 2006 and 2008 before rising again by 20% between 2008 and 2010. The lack of a strong mental model relative to the social implications of science and society may account for this variability.

Finally, even though all four groups between 2002 and 2010 increased in their support for embryonic stem cell research, the gap in views between Scientific Optimists and Scientific Pessimists remained relatively the same. In 2002 the difference in views between the two groups was 27%; eight years later, it was also 27 points.

## Discussion

Consistent with past studies, our findings indicate that political partisanship had a significant effect on public opinion in the stem cell debate and that the influence of partisanship was greatest among the better educated. In an era of extreme polarization, this finding is reflective of the heavy investment across issues by U.S. political leaders and activists in making sure that the public is aware of the diverging policy positions of the two major political parties and their candidates. In correlation with this investment, better-educated Democrats and Republicans, who are typically more attuned to these message strategies, over time tend to align their own opinions accordingly. In this case, the debate over stem cell research is no exception to broader trends related to polarization, but instead a leading example of these dynamics at work.

Yet our regression results also indicate that more so than partisanship, ideology, religious identity, self-rated knowledge, or abortion views, an individual's beliefs about science and society had the strongest influence on support for embryonic stem cell research. Moreover, the findings from our principle components analysis indicate that unique segments of the public differ substantially in how they perceive the social implications of science and technology and that these groups are not easily defined by their political partisanship.

“Scientific optimists”—who tend to be highly educated, financially secure, and disproportionately white—believe strongly in the link between science and social progress and tend to be overwhelmingly supportive of scientific advances such as embryonic stem cell research. In contrast, “Scientific pessimists”—who on average score much lower in terms of educational attainment and income—have reservations about the moral boundaries that might be crossed by scientists and are more likely to oppose advances in biomedical research and related fields. A third segment, the “Conflicted,” view science in both optimistic and pessimistic terms. They are in many ways socially similar to Scientific Pessimists in their background, but tend to be older than members of other segments. Though they are ambivalent about the impacts of science on society, they appear open to accepting the arguments of scientists and advocates who emphasize the benefits of research. A fourth segment, the “Disengaged,” appear to lack a well-formed mental model for how science and technology might impact society and consider themselves less informed. For this group, absent strong schema about science and society and limited subject-specific knowledge, they are likely to be the most susceptible to shifts in opinion as they rely on partisan cues and focusing events rather than strongly formed schema about science and society to guide their judgments.

For all of the identified audience segments, how they apply their generalized beliefs about science and society to a specific issue will be in part a function of the selective frames and storylines found in news coverage, entertainment programming, popular culture, social media and face-to-face conversations [9], [14], a process that should be further investigated in future studies. In addition, researchers should look to improve upon the measurement of science and society schema by employing more extensive multi-item scales and by profiling more closely the social composition of each audience segment along with their communication behaviors and trusted sources of information. This research would also benefit from including direct measures of specific forms of science- and policy-related knowledge rather than relying on self-ratings.

For social scientists, focusing on deeper attitudinal distinctions relative to science and society also allows for comparisons across countries where partisan or ideological categories similar to those in the U.S. do not exist. Even in the U.S., political leaders on the left and the right seldom split into easily identifiable “pro-science” and “anti-science” factions. Consider that in 2009, when President Obama expanded federal funding for research, he chose not to provide funding for stem cells derived from cloned embryos, going against the requests and recommendations of scientists [38]. Public opinion surveys show that since 2002, less than a majority of Americans have favored medical or therapeutic cloning procedures [22], indicating that the public holds intuitive reservations about some areas of biomedical research, reservations that transcend partisan and ideological differences. Moreover, among liberal intellectuals and feminists, therapeutic cloning and the creation of embryos for research purposes have been the target of criticism, as these advocates warn of using human life for instrumental or market purposes and of the possible exploitation of egg donors [39] [40]. Similarly, Canada and Germany each have stricter limits on embryonic stem cell research than the U.S., despite a more liberal and secular political culture [32] [41].

Research in countries other than the U.S. also indicates strong public reservations about embryonic stem cell research when it is conducted by private companies rather than publicly funded university scientists [42]. Studies in the U.S. have yet to carefully explore public judgments about the privatization, patenting, and commercialization of stem cell related therapies, but it is likely that when these issues become the subject of news attention and political debate, reservations about privatization and control are likely to transcend partisan differences. How different segments of the public may respond to debates over commercialization and privatization is especially relevant given that bioethicists warn of an intensifying “cycle of hype” in the claims made about biomedical research [43], [44]. Specific to stem cell research, they have called for a more “honest acknowledgement of the expected therapeutic benefits and the timelines to achieving them.” [26] As uncertainty about the clinical applications of embryonic stem cell research lingers and the timeline to such applications remains in doubt, then at risk is the broader public's trust in science as an institution.

Our findings also suggest several important implications for public communication and engagement. In the future, as new debates specific to biomedicine, neuroscience, nanotechnology and other fields emerge, if scientists and their institutions focus exclusively on the potential for partisan and ideological differences they will be potentially distracted by relatively simplistic left versus right distinctions that will vary considerably in relation to media attention, elite cues, and specific policy proposals. In contrast, the science-related schema identified in this study are likely to be more stable over time, less volatile, and have greater predictive power in assessing public opinion and in pro-actively addressing sources of contention or controversy.

Given the trend towards group polarization in society generally, the propensity towards hype and the importance of public trust, additional research that further develops and tests the validity of the identified schema-related audience segments will also be of important use to government agencies, universities, research institutes and other organizations. As has been shown in climate change communication research, profiling and segmenting the public in more precise and valid ways than partisanship and ideology will allow these institutions to tailor and make more effective their communication activities. These activities should include contextualizing information in a manner that is personally relevant and understandable, working with and by way of trusted information providers and opinion-leaders, and investing in media forums that broker cross-cutting dialogue and debate [45]–[47].

Consider the challenge of effectively engaging the audience segment of Scientific Pessimists, a segment that comprises nearly 1 out 4 U.S. adults. Efforts aimed at simply better informing or educating this segment about scientific advances as they become politically contested and the subject of media debate are unlikely to meaningfully change perceptions. In fact, previous studies suggest that such efforts may only serve to reinforce opposition [3]–[8]. Consistent with these studies, our findings show that in fact it is the best educated among this group who are likely to be the most opposed to advances in biomedical research, the most receptive to the arguments made by political opponents to such research, and the most dismissive of those advocating on behalf of research.

Outreach efforts by scientists and expert institutions, therefore, need to draw upon audience research in designing initiatives that directly address the nature of these moral and ethical concerns and in doing so partner with opinion-leaders who are trusted by this segment and others. Examples might include carefully designed and evaluated Web-based resources and documentary films or online multimedia platforms combined with localized public forums that blend discussion of science with that of various ethical, legal and religious perspectives. These efforts which, for example, could be sponsored via partnerships among government agencies, scientific societies, media organizations, foundations, universities, research institutions, faith-based organizations, and/or minority groups would be designed to establish an ongoing dialogue with those segments of the public who have the strongest reservations about the impacts of science on society [9], [11], [48]–[51].
